# Binary Associative Memories as a Benchmark for Spiking Neuromorphic Hardware

**DOI:** 10.3389/fncom.2017.00071

**Published:** 2017-08-22

**Authors:** Andreas Stöckel, Christoph Jenzen, Michael Thies, Ulrich Rückert

**Affiliations:** Cognitronics and Sensor Systems, Cluster of Excellence Cognitive Interaction Technology, Faculty of Technology, Bielefeld University Bielefeld, Germany

**Keywords:** neuromorphic hardware, spiking neural networks, benchmark, associative memory

## Abstract

Large-scale neuromorphic hardware platforms, specialized computer systems for energy efficient simulation of spiking neural networks, are being developed around the world, for example as part of the European Human Brain Project (HBP). Due to conceptual differences, a universal performance analysis of these systems in terms of runtime, accuracy and energy efficiency is non-trivial, yet indispensable for further hard- and software development. In this paper we describe a scalable benchmark based on a spiking neural network implementation of the binary neural associative memory. We treat neuromorphic hardware and software simulators as black-boxes and execute exactly the same network description across all devices. Experiments on the HBP platforms under varying configurations of the associative memory show that the presented method allows to test the quality of the neuron model implementation, and to explain significant deviations from the expected reference output.

## 1. Introduction

Neuromorphic hardware systems promise to simulate large-scale spiking neural networks at or above biological realtime with a fraction of the energy requirements of supercomputer simulations. As such, they are important tools for research regarding the computational principles of brain-like networks (Hasler and Marr, 2013). As part of the Human Brain Project (HBP, Markram 2012), two neuromorphic platforms are being developed: the digital many-core SpiNNaker system (Furber et al., 2013; Painkras et al., 2013), and the mixed-signal BrainScaleS physical model system (Schemmel et al., 2010; Petrovici et al., 2014). However, there is no universal, well assessable measure to characterize the performance of neuromorphic hardware, as it is common for classical supercomputers, e.g., Feng and Cameron (2007). This can in part be ascribed to the vast architectural differences between neuromorphic systems. Digital simulators such as SpiNNaker are theoretically capable of solving any mathematical neuron model with high precision. Mixed-signal physical models such as BrainScaleS offer emulation of neural networks at several magnitudes speed-up compared to biological realtime, yet are limited in their adaptability and prone to deviations caused by the manufacturing process and noise in the analog circuits. Benchmarks for neuromorphic hardware must not only account for the raw runtime and energy efficiency, but also for the achieved simulation accuracy under a certain workload.

In this paper we propose the amount of information stored in a regularly structured feed-forward binary neural associative memory (BiNAM) as a benchmark indicator for neuromorphic hardware systems. The BiNAM has been studied by Kohonen, Steinbruch, Willshaw, Palm, and others since the 1970s as an efficient method for pattern mapping, completion, and fault tolerant information retrieval in technical systems and as a model for associations in biological neural networks (Steinbuch, 1961; Willshaw et al., 1969; Kohonen, 1977; Palm, 2013). Furthermore, activity of different neurons belonging to the same pattern can be seen in the light of Hebb's theory of cell assemblies (Hebb, 1949), which can be interpreted as memory states (mental objects) in the cortex (Lansner, 2009).

For multiple reasons, the BiNAM is of particular interest as a neuromorphic hardware benchmark. Foremost, the theoretical properties of the memory are well understood when implemented as a network of non-spiking McCulloch-Pitts neurons (McCulloch and Pitts, 1943). This includes measures such as the expected storage capacity of the memory (Palm, 1980; Rückert and Surmann, 1991; Schwenker et al., 1996). An implementation of the threshold function found in McCulloch-Pitts neurons on a spiking substrate requires careful tuning of neuronal and synaptic parameters and is susceptible to deviations in spike timings. The spiking BiNAM correspondingly tests how well the mathematical neuron model, parameters and spike timings are reproduced by the target platform. Furthermore, the binary neural associative memory network is relatively simple, regularly structured, and arbitrarily scalable. Simplicity, e.g., the lack of feedback and inhibitory neurons, facilitates mapping to experimental systems with still incomplete software stacks and mapping routines, and thus allows to gather results during their development. The highly regular structure of the associative memory network potentially allows the localization of anomalies in the hardware system and to infer the cause of unexpected deviation from expected behavior. Scalability is important for full utilization of the target platform, from single-board dissemination up to large-scale hardware systems. With these properties, our benchmark is a candidate for low-level examination and comparison of neuromorphic platforms, and, by informing about deviations from theoretical neuron models, an aid to end-users designing networks. Nevertheless, it must be emphasized that the benchmark is neither intended to replace tests for specific hardware features, nor does it directly quantify the ability of a platform to simulate complex, biologically plausible, and functional neural networks.

So far, neuromorphic hardware benchmarks focus on individual hardware systems instead of a generic black-box approach. For example, several benchmarks exist for the SpiNNaker platform, including an examination of neuron accuracy (Sharp and Furber, 2013), an in-depth power analysis of the SpiNNaker chip (Stromatias et al., 2013), as well as an implementation of the neural engineering framework (Mundy et al., 2015), and proof of concepts, such as a pre-trained deep-belief network for the MNIST handwritten digit dataset (Stromatias et al., 2015). More recent experiments (van Albada et al., 2016) demonstrate the ability of SpiNNaker to simulate large-scale biological models such as a cortical microcircuit, while closely reproducing the results of the NEST software simulator (Gewaltig and Diesmann, 2007).

Apart from specialized tests aiding the design of internal software components (Ehrlich et al., 2010), BrainScaleS, its emulator ESS (Brüderle et al., 2011), and the small-scale precursor system Spikey (Brüderle, 2009), were evaluated by analyzing, among others, an attractor network, a synfire chain and a self-sustained asynchronous irregular activity model (Brüderle et al., 2011; Pfeil et al., 2013; Petrovici et al., 2014). In these publications, results are analyzed in great detail and compared to simulations with pure software simulators. Yet, they lack benchmark measures or indicators for automatic evaluation and it is not inherently clear how the different implementations of the same network model (e.g., the synfire chain) should be compared.

While the aforementioned studies focus on first proof of concepts tailored to individual platforms, a benchmark testing a variety of neuromorphic platforms has been developed in Diamond et al. (2015). Here, the SpiNNaker, Spikey, and GeNN (a GPU-based simulator, Yavuz et al. 2016) platforms are compared with a bio-inspired network of the insect olfactory system. In contrast to our approach, each implementation is hand tuned to the individual platform (Schmuker et al., 2014). This allows to answer the question how well a certain task can possibly be solved on a given system. In this paper however, we attempt to assess the quality of the entire hard- and software stack available to end-users by executing exactly the same network across all platforms in one set of experiments (Sections 3.3 and 3.4), and scaling the network to platform-dependent high-workload sizes in another (Section 3.1).

The remainder of this paper is structured as follows: in Section 2 we present the BiNAM, its spiking neural network implementation, describe the target platforms, and outline the overall benchmark procedure. The actual experiments, consisting of a benchmark run with high workload, a data parameter sweep, a neuron parameter sweep, and a power efficiency analysis, are given in Section 3, followed by a concluding discussion in Section 4.

## 2. Methods

The proposed neuromorphic hardware benchmark is a translation of a theoretical associative memory model to a time-dynamic spiking neural network. In this section we review the theoretical model and the corresponding network topology, followed by discussions on how information is coded over time, how neuron parameters are selected, which characteristics of the target platforms have to be taken into account, and finally, which experimental protocol is used to analyze the network performance.

### 2.1. Binary neural associative memory (BiNAM)

Associative memories typically possess two modes of operation: training and recall. In the training phase, the memory stores key-value pairs ${\vec{x}}_{k}\mapsto {\vec{y}}_{k}$. Given an arbitrary input vector $\vec{x}$, the recall operation for an optimal associative memory is defined as (Gerstner et al., 2014)

(1) $$\begin{array}{l} f^{*}(\vec{x})={\vec{y}}_{k}\text{ where }k=\arg\;\underset{k^{\prime }}{\min}\|{\vec{x}}_{k^{\prime }}-\vec{x}\|. \end{array}$$

In other words, an optimal associative memory returns the ${\vec{y}}_{k}$ associated to the ${\vec{x}}_{k}$ best matching the current input $\vec{x}$. This function can be interpreted as resilient content-addressed memory access, or, assuming auto-association (${\vec{x}}_{k}={\vec{y}}_{k}$) and incomplete $\vec{x}$, as pattern completion. Both are considered key functions of biological brains (Palm, 2013).

The BiNAM is a particular implementation of an associative memory (Willshaw et al., 1969; Palm, 1980). As elaborated in Palm (1980), it stores associations in a binary matrix *M* ∈ *B*^*m*×*n*^ set to a superposition of outer products of ${\vec{x}}_{k}$ and ${\vec{y}}_{k}$

(2) $$\begin{array}{l} M=\overset{N}{\underset{k=1}{\vee }}{\vec{x}}_{k}\cdot {\vec{y}}_{k}^{T}\text{ with }{\vec{x}}_{k}\in {𝔹}^{m},{\vec{y}}_{k}\in {𝔹}^{n}, \end{array}$$

where ∨ denotes the logical “or” operator and *N* the number of samples. This training operation can be interpreted as Hebbian learning (Hebb, 1949), where individual synaptic weights (here: matrix entries (*M*)_*ij*_) grow stronger in the presence of synchronous pre- and post-synaptic activity (Palm, 2013). Recall is defined as multiplication of $\vec{x}$ with *M* followed by a threshold operation (Heaviside function)

(3) $$\begin{array}{l} {\left({\vec{y}}_{k}^{\;\prime }\right)}_{i}={\left(f(\vec{x})\right)}_{i}=\{\begin{array}{ll} 1 & {\left({\vec{x}}^{T}\cdot M\right)}_{i}\geq \;\|\vec{x}{\|}_{1}\\ 0 & \text{otherwise} \end{array}\cdot \end{array}$$

Generally, the recall operation *f* is not optimal in the sense of f^*^ in Equation (1): ${\vec{y}}_{k}^{\prime }$ may contain additional entries equal to one not in the trained ${\vec{y}}_{k}$ (false positives). Mathematically, the opposite is not possible (there are no false negatives, “ones” in ${\vec{y}}_{k}$ missing in ${\vec{y}}_{k}^{\prime }$) (Palm, 1980). Practically, false negatives are introduced if the recall process is implemented on a substrate such as a spiking neural network with inappropriate neuron parameters, or due to stochastic processes in simulators or neuromorphic hardware.

In order to simplify mathematical analysis of the memory, we constrain the number of non-zero entries in all input and output vectors to constant numbers $||{\vec{x}}_{k}|{|}_{1}=c$ and $||{\vec{y}}_{k}|{|}_{1}=d$. Assuming statistical independence of all vectors ${\vec{x}}_{k}$ and ${\vec{y}}_{k}$, and given false positive and negative counts α_*k*_, β_*k*_ for each sample *k*, the information *I* in bits recallable from the memory is given as (Rückert and Surmann, 1991)

(4) $$\begin{array}{l} I=\sum_{k=1}^{N}\log_{2}\left(\begin{array}{c} n\\ d \end{array}\right)-\log_{2}\left(\begin{array}{c} \alpha_{\text{k}}+d-\beta_{\text{k}}\\ d-\beta_{\text{k}} \end{array}\right)\\ \;-\log_{2}\left(\begin{array}{c} n-\alpha_{\text{k}}-d+\beta_{\text{k}}\\ \beta_{\text{k}} \end{array}\right)\cdot \end{array}$$

The expected average number of false positives per sample $\tilde{\alpha }$ can be approximated as (Palm, 1980)

(5) $$\begin{array}{l} \tilde{\alpha }=(n-d)\cdot {\left(1-{\left(1-\frac{c\cdot d}{m\cdot n}\right)}^{N}\right)}^{c}\cdot \end{array}$$

Combining $\alpha_{k}=\tilde{\alpha }$ and β_*k*_ = 0 with Equation (4) yields an approximate formula for the information capacity *I* under optimal conditions. Maximizing *I* with respect to the number of samples allows to approximate the optimal number of samples that should be stored in the memory. The highest capacity is reached for sparse data with small *c*, *d* which are of the order of log(*n*) and log(*m*), respectively (Palm, 1980).

### 2.2. Spiking neural network implementation

**Figure 1:**
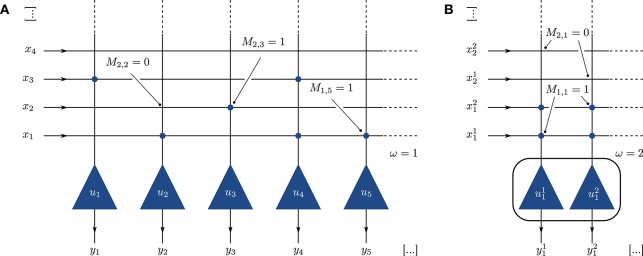
**(A)** Implementation of the BiNAM as a neural network. Each output vector component is represented by a single neuron *u*_1_, …, *u*_*n*_ (triangles). Synaptic connections between input and the output (circles) are created according to the trained memory matrix *M*. **(B)** Single column of a network with population size ω = 2. The output components are represented by ω neurons, each receiving ω input signals per set bit in the input vector.

The spiking BiNAM topology is a straight-forward translation of the McCulloch-Pitts network described in Palm (1980). It consists of a single layer of neurons, in which each individual neuron represents one output component *j*. A synaptic connection between input signal *i* and neuron *j* is established if the corresponding entry in the trained storage matrix (*M*)_*ij*_ is set to one (Figure 1A).

Ones in the input vectors $\vec{x}$ are encoded as a burst of *s* spikes with inter-spike interval Δ*t* = 2 ms. This Δ*t* is relatively small, but has been found to be not unusual for small bursts of 4-5 spikes of pyramidal cells in visual cortex (Gray and McCormick, 1996). Additionally, Gaussian jitter with standard-deviation σ_*t*_ = 2 ms is added to all spike times, with the same sequence of random numbers being used for equal experimental setups. We chose these values to attain an input rate of approximately 500 s^−1^ per synapse, resulting in a combined peak input rate of 2.000 s^−1^ for *c* = 4 simultaneously active inputs. This is a small value compared to the combined input rate of a cortical neuron, which can be roughly estimated to be about 10^4^ s^−1^ (Braitenberg and Schüz, 1998). Correspondingly, we expect our network to be well realizable on all tested neuromorphic hardware platforms. To eliminate the risk of inter-pattern interference, a new input sample ${\vec{x}}_{k}$ is presented to the network every 100 ms, which guarantees the recovery of every neuron to a resting state. We run our benchmark for two burst sizes *s* = 1 and *s* = 4, where the former describes a burst-less representation and the latter value is chosen to have maximal neuron activity only in the first ten milliseconds, to further assure the recovery to resting potential in the inter-pattern interval.

Output spike trains are decoded into a binary representation by counting the number of output spikes for each neuron (or neuron population of size ω, see below) during each 100 ms sample presentation period. In case the output spike count of the *j*-th neuron or neuron population exceeds ω during a presentation period, the corresponding output vector component ${\left({\vec{y}}_{k}^{\prime }\right)}_{i}$ is set to one, otherwise it is set to zero.

To increase the network robustness on platforms which encounter spike loss, we alternatively replace each neuron with a small neuron population of size ω. Each input and output component is represented by ω independent signals. Connections are performed using an all-to-all connector, resulting in ω^2^ synaptic connections for each one-entry in *M* (Figure 1B). This representation allows to compensate for the loss of input spikes and variability in the neuron parameters, as the information carried in a single spike is reduced. Furthermore, and as elaborated in Section 2.3, the use of neuron populations widens the potential space of neuron parameters which implement the desired neuronal threshold behavior. Note that this procedure requires more neurons per output component and therefore decreases the overall memory capacity achievable on a fixed-size network.

In order to use the entire Spikey system, ω is restricted to common divisors of 256 and 384 (e.g., 2, 4, 8, 16; see Section 2.4). To balance between a reasonable memory size and a high multiplicity, we select both ω = 1 (no neuron multiplicity) and ω = 4.

As our neuron model we choose a linear integrate-and-fire point neuron with conductance-based synapses with exponential decay (IfCondExp). This decision is based on our intent to execute the same network on all target platforms, which limits neuron model and parameter selection to the smallest common denominator, in our case the Spikey neuromorphic system (Section 2.4). With the exception of a single experiment, our selected neuron parameters fall into the parameter range supported by Spikey (see also Table 1).

**Table 1:** Neuron parameter sets *I*–*III* used in this paper.

**Neuron parameters**									
**Parameter**			**Default parameters**			Spikey^*^parameters			**Spikey range**
**Name**	**Symbol**	**Unit**	**I**	**II**	**III**	**I**	**II**	**III**	
Resting potential	*V*_rest_	[mV]	−80.0	−80.0	−80.0	−70.0	−75.0	−80.0	−80.0 to −55.0
Threshold potential	*V*_th_	[mV]	−57.0	−64.7	−62.0	−59.0	−55.0	−55.0	−80.0 to −55.0
Reset potential	*V*_reset_	[mV]	−80.0	−80.0	−80.0	−80.0	−80.0	−80.0	−80.0 to −55.0
Refractory time^b^	τ_ref_	[ms]	1.0	1.0	1.0	1.0	1.0	1.0	1.0
Leak conductance	*g*_leak_	[nS]	20.0	20.0	89.0	39.0	37.0	40.0	20.0 to 40.0
Membrane cap.^b^	*C*_m_	[nF]	0.2	0.2	0.2	0.2	0.2	0.2	0.2
Weight^c^	*w*	[nS]	10.0	1.0	1.0	6.0	3.0	1.0	0.0 to 15.0
Reversal potential^b^	*E*_exc_	[mV]	0.0	0.0	0.0	0.0	0.0	0.0	0.0
Time constant^a^^,^^b^	τ_exc_	[ms]	2.0	2.0	2.0	5.0	5.0	5.0	2.0

*Parameter set I for s = 1, ω = 1; II for s = 1, ω = 4 and s = 4, ω = 1; III for s = 4, ω = 4. Spikey parameters adapted form (Brüderle, 2009) and corresponding source code. See Section 2.4 regarding Spikey parameter fitting*.

a *Spikey range is experimentally determined*;

b *Not user-definable on Spikey via PyNN*;

c *Synaptic weights discretized with 4 bit resolution on Spikey*.

### 2.3. Parameter selection

A common goal of neuron parameter optimization in neuroscience is to fit a model response to electrophysiological measurements (Brillinger, 1988; Bhalla and Bower, 1993; Gerstner, 2008; Gerstner et al., 2014). Techniques—with varying degrees of applicability—include parameter space exploration, gradient descent, bifurcation analysis, and evolutionary algorithms (Prinz, 2007).

Parameter optimization in the case of the spiking binary neural associative memory differs from common neuroscientific approaches insofar as we do not fit the neuron response to a given spike train, but optimize the number of output spikes in response to a certain stimulus. Precise output spike timings are of secondary concern. Correspondingly, we assess the quality of neuron and synapse parameters θ in terms of a joint probability

(6) $$\begin{array}{l} P(n(I_{1})={\tilde{n}}_{1},\ldots ,n(I_{\ell })={\tilde{n}}_{\ell }|I_{1},\ldots ,I_{\ell },\theta )\\ \;=\prod_{i=1}^{\ell }P(n(I_{i})={\tilde{n}}_{i}|I_{i},\theta ), \end{array}$$

where $P(n(I_{i})={\tilde{n}}_{i}|\text{​}I_{i},\text{​}\theta )$ is the likelihood of a neuron to produce ñ_*i*_ output spikes given a time- and membrane potential-dependent input current *I*_*i*_(*t, u*). In our particular setup the input current is generated by a spike train arriving at a conductance based synapse with exponential decay.

As elaborated above in Sections 2.1 and 2.2, each neuron in the spiking BiNAM implementation must implement a threshold function. Such a function can be specified in terms of the above framework with ℓ = 4 objectives. For both *I*_1_ = 0 and an input *I*_2_ modeling *s* · ω · (*c*−1) input spikes, the neuron should produce ñ_1, 2_ = 0 output spikes. In contrast, for stimuli *I*_3, 4_ modeling *s* · ω · *c* and *s* · ω · (*c*+1) input spikes, the neuron should output a single burst consisting of ñ_3, 4_ = *s* output spikes. Input spike times are selected according to the constraints laid out in Section 2.2.

An empirical approach to estimating the probabilities in Equation (6) is to sample noisy stimuli *I*_*i*_ and measure the actual output spike counts for each sampled input. Then, $P(n(I_{i})={\tilde{n}}_{i}|\text{​}I_{i},\text{​}\theta )$ is given as the fraction of trials in which the desired output spike count is produced. This method is particularly useful for manual parameter fine-tuning. It is less suited for automated parameter optimization, since the joint probability is zero in large portions of the parameter space, providing neither a gradient for gradient descent, nor potential for random improvement in evolutionary algorithms. Furthermore, this approach requires hundreds of trials to provide good estimates and is thus relatively slow (Stöckel, 2015).

An alternative approach to the estimation of the above probabilities is the *fractional spike count* measure 𝔮. This measure describes both the number of output spikes (integral part) and the likelihood of an additional output spike being produced 𝔭 (fractional part). 𝔭 is defined in terms of a one-dimensional bifurcation analysis with respect to a perturbation current *j*, which either increases or decreases the output spike count. Let *j*^+^ denote the minimal excitatory current which increases the number of output spikes relative to the unperturbed simulation

(7) $$\begin{array}{l} j^{+}(I|\theta )=\min\{j|n(I+j|\theta )>n(I|\theta )\}\cdot \end{array}$$

For 𝔫(*I* ∣ θ) > 0, the current *j*^−^ is the minimal inhibitory current which decreases the output spike count

(8) $$\begin{array}{l} j^{-}(I|\theta )=\min\{j|n(I-j|\theta )>n(I|\theta )\}\cdot \end{array}$$

In case 𝔫(*I* ∣ θ) = 0, the current *i*^−^ is defined as the minimal inhibitory current which suppresses all neuronal activity to a point where the membrane potential *u*(*t, I* ∣ θ) does not exceed the resting potential *v*_rest_ at any point in time

(9) $$\begin{array}{l} j^{-}(I|\theta )=\min\{j|u(t,I-j|\theta )\leq v_{\text{rest}}\forall t\}\cdot \end{array}$$

Given these perturbation currents, 𝔭 is defined as

(10) $$\begin{array}{l} p(I|\theta )=\frac{j^{-}(I|\theta )}{j^{+}(I|\theta )+j^{-}(I|\theta )}\cdot \end{array}$$

**Figure 2:**
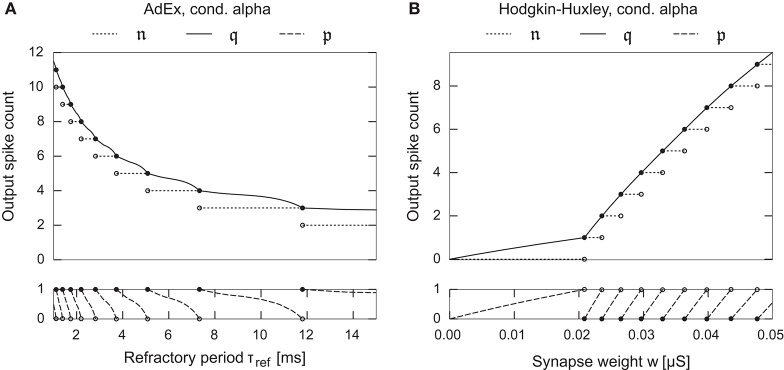
Examples depicting the fractional spike count in Equation (10) as a one dimensional function over a neuron/synapse parameter for two different neuron models. **(A)** Adaptive Exponential neuron, Brette and Gerstner (2005). **(B)** Hodgkin-Huxley type neuron model with kinetics as fitted by Traub and Miles (1991). Both neurons possess a single conductance based synapse with alpha-function shaped dynamics. The fractional spike count smoothly interpolates between the corner-points of the spike count function, facilitating gradient based parameter optimization.

Assuming a monotonic change in spike counts with respect to *j*, this measure can be calculated to high precision with a binary search. As shown in Figure 2, the method results in a smooth gradient when varying neuron parameters, which facilitates gradient-based optimization even for more detailed synapse and neuron models as those used in the remainder of this paper. We derive the probabilities in Equation (6) by replacing the random variables 𝔫(*I*_*i*_) with $𝔮\left(I_{i}\right)-\frac{1}{2}$ and assuming a heavy-tailed Student's *t* distribution. We then optimize Equation (6) with respect to the threshold-function objectives introduced above using the Nelder-Mead method with random restart (Nelder and Mead, 1965; Press et al., 2007). Since this approach only results in an approximation of the goal function in Equation (6), we manually fine-tune the resulting neuron parameter estimate according to the more accurate, yet slow, empirical probability estimate.^1^ Final optimized parameters as used in the experiments are shown in Table 1.

### 2.4. Target platforms

As a reference platform we use the *NEST* software simulator in version 2.10 (Gewaltig and Diesmann, 2007; Bos et al., 2015). Individual network simulations were performed single-threaded on an Intel Core i7-4710MQ processor. The IfCondExp model used in our experiments is solved by NEST with an adaptive fourth order Runge-Kutta-Fehlberg integrator with fifth order error estimate and 10^−3^ absolute target error. Spike propagation and threshold handling are synchronously performed with a user-defined time-step of 0.1 ms. For a small set of experiments we manually patched the NEST source code to implement a naïve Euler integrator^2^, which allows to analyze possible discrepancies to SpiNNaker simulations (see below). However, note that our integrator does not implement any of the optimizations present in SpiNNaker (Rast et al., 2010).

The *SpiNNaker* system (Furber et al., 2013) is a digital many-core architecture which integrates 18 general-purpose processors on a single chip, along with multi-link routers for inter- and intra-chip spike event propagation. Depending on the network topology and neuron model, up to one thousand neurons can be simulated on a single core at one thousand update steps per second (Painkras et al., 2013). The IfCondExp model as implemented in the sPyNNaker software package in version 2016.001 uses a 32-bit fixed-point state space representation and an Euler integrator to solve the model equations (Rast et al., 2010). We benchmark the system performance at biological real-time with a time step of 1.0 ms. This mode is of special importance, as it allows SpiNNaker to be used as a platform for realtime neuro-robotics. A possible bottleneck of the SpiNNaker platform is network congestion caused by limited connection bandwidth and routing table entries. This may result in spike events being dropped or delivered too late to the target neuron. For our experiments we use a four-chip stand-alone SpiNNaker board.

The *BrainScaleS* physical model system (Schemmel et al., 2010; Petrovici et al., 2014) implements the Adaptive Exponential (AdEx) neuron model (Brette and Gerstner, 2005) as an analog VLSI circuit. The system integrates entire silicon wafers of *HICANN* chips, each containing two blocks of 256 analog AdEx neurons. The model runs at a speed-up factor of 10^4^ compared to biological real-time. In order to emulate the simpler IfCondExp neuron model, parts of the AdEx model can be deactivated. Each neuron possesses 224 synapse circuits^3^, and up to 64 neuron circuits can be combined into a single logical neuron with improved stability and increased input count. Synaptic weights are discretized to 4-bit resolution and neuron parameters are stored with an effective 10-bit resolution. In our experiments we use a logical neuron size of four neuron circuits. Spike events are transferred between synapse circuits, individual HICANNs, and across wafer boundaries via a digital interconnect (Fieres et al., 2008; Scholze et al., 2011), leading to similar restrictions as for the SpiNNaker platform. In addition, possible deficiencies may include an imperfect realization of the mathematical neuron model, neuron-to-neuron variations, trial-to-trail variations (due to analog reconfiguration), as well as non-deterministic fluctuations in the model state and parameters during emulation. At the time of writing, the hardware system itself could not be used for our experiments. Instead, we run our simulations on the *BrainScaleS-ESS*^4^ (executable system specification, short *ESS*), which simulates the digital communication infrastructure (including bandwidth constraints and limitations on spike processing) in addition to the actual hardware neuron (with discrete parameters, range restrictions, and the option to impose noise on synaptic weights). The simulator and the hardware system share the same software-frontend and processing steps such as the conversion from biological to hardware parameters as well as the mapping algorithm (Petrovici et al., 2014). Compared to the real hardware, we expect the benchmark results to be slightly better in simulation, since not all potential sources of noise and variability present in the actual physical system are modeled. However, since the communication infrastructure and mapping are implemented in high detail, the ESS is an excellent tool to analyze digital communication bottlenecks and discrepancies between the user-defined network and its hardware realization, such as the omission of synaptic connections due to hardware constraints.

The *Spikey*^5^ single chip system (Pfeil et al., 2013) contains a predecessor of the HICANN chip used in BrainScaleS and implements a physical VLSI model of the IfCondExp model with limited flexibility in neuron and synapse parameters (Table 1). The Spikey chip contains 384 neuron circuits with 256 synapses each. As with BrainScaleS, simulations are executed at 10^4^ times biological realtime and synaptic weights discretized to four bits. Due to the analog nature of the system, membrane and synapse circuits only provide an approximation of the mathematical neuron model along with fluctuations in parameters and state. This results in a non-deterministic behavior of single neurons, and in consequence the entire network, if not specifically designed to compensate for such deviations (Bill et al., 2010). Note that the excitatory synaptic time constant τ_exc_ is not exposed to users via PyNN, and thus treated as constant in this study. Its actual value depends on various factors, including the system calibration and the particular network connectivity and parameters (Brüderle, 2009). Since the mathematical neuron model used in the parameter optimization process requires an estimate of τ_exc_, we experimentally measured a typical excitatory post-synaptic potential (EPSP) produced by Spikey and fitted the response of the mathematical neuron model with varying degrees of freedom. Consistently, lowest approximation errors were achieved for τ_exc_ ≈ 2 ms. See supplemental material for details.

### 2.5. Benchmark procedure

Given data parameters *m*, *n*, *c*, *d* (Section 2.1), we generate *N* independent and uniformly distributed random (yet reproducible) heteroassociative training data pairs ${\vec{x}}_{k}\mapsto {\vec{y}}_{k}$. The data generator additionally imposes uniqueness on the vectors and ensures minimum variance across the entries of the sum of the first *N*′ vectors for any *N*′ ≤ *N*. This guarantees a balanced workload, yet technically violates sample independence assumed to derive Equation (4). In practice, any dependence caused by these additional constraints is small as long as reasonably large *m*, *n* are selected. Subsequently, we calculate the matrix *M*, the theoretical information baseline *I*_th_, and the average false positives per sample ${\bar{\alpha }}_{th}$ according to Equations (2) to (4).

The network and input spike trains are constructed as described in Section 2.2 and subsequently executed on the target platform. In the special case of parameter sweeps on SpiNNaker, multiple independent networks are multiplexed and executed in parallel. In addition, networks are executed in random order to minimize correlations between individual runs on the Spikey system. The number of networks simulated in parallel depends on the available hardware resources. In case of software simulations, the networks are simulated in independent processes. We measure the execution time *t* including platform specific setup/teardown. The neuronal output is recorded and then decoded into the binary memory response ${\vec{y}}_{k}^{\prime }$, resulting in the actual retrievable information *I* and average false positive and negative counts $\bar{\alpha }$, $\overset{-}{\beta }$. Comparison with the theoretical baseline yields a set of normalized measures

(11) $$\begin{array}{l} I_{\text{n}}=I/I_{\text{th}},\;{\bar{\beta }}_{n}=\bar{\beta }/d,\\ {\bar{\alpha }}_{n}=\{\begin{array}{ll} \bar{\alpha }/{\bar{\alpha }}_{th}-1, & \text{if }\bar{\alpha }\leq {\bar{\alpha }}_{th}\\ \left(\bar{\alpha }-{\bar{\alpha }}_{th})/(n-d-{\bar{\alpha }}_{th}\right) & \text{otherwise}. \end{array} \end{array}$$

Here, values $I_{\text{n}}=1,\;{\overset{-}{\alpha }}_{\text{n}}=0$, and ${\overset{-}{\beta }}_{\text{n}}=0$ correspond to a perfect reproduction of the theoretical model. The false positive count ${\overset{-}{\alpha }}_{\text{n}}$ is scaled to [−1, 1], with positive and negative values corresponding to a shortage or surplus in false positives compared to the expected values. Note that *I*_n_ is not the amount of recallable information relative to the number of bits in the storage matrix *M*, which has a theoretical maximum at ln(2) ≈ 69% (Palm, 1980). Instead, the normalized information *I*_n_ is relative to the theoretically expected recallable information *I*_th_ for each individual dataset. It should be regarded as the relevant benchmark indicator, the other values are auxiliary measures. However, note that the achievable *I*_n_ does not only depend on the quality of the simulator, but also on the selected neuron parameters, mandating to ensure that *I*_th_ can actually be reached for the chosen parameter set in a reference simulator.

Note that *I*_n_ is only meaningful as a benchmark metric as long as the memory matrix *M* is not saturated with one-entries. Otherwise, vectors recalled from a random memory matrix—or equivalently, a badly behaved target platform—may lead to information values *I* larger than the theoretical optimum *I*_th_. Assume a random memory matrix *M* with *P*((*M*)_*ij*_ = 1) = 0.5. The average false-positive ${\overset{-}{\alpha }}_{\text{rand}}$ and false-negative ${\overset{-}{\beta }}_{\text{rand}}$ counts in a vector ${\vec{y}}^{\prime }$ recalled from such a random matrix can be estimated as

(12) $$\begin{array}{l} \;℘=P\left({\left({\vec{y}}^{\prime }\right)}_{i}=1\right)\approx \sum_{k=c^{\text{th}}}^{m}B\left(k;m,\frac{c}{2\cdot m}\right),\\ {\bar{\alpha }}_{rand}=\left(n-d\right)\cdot (1-℘),\;{\bar{\beta }}_{rand}=d\cdot ℘, \end{array}$$

where *B*(*k*; *n, p*) is the probability mass function of a binomial distribution, and *c*^th^ is the threshold value encoded in the network (in most cases *c*^th^ = *c*). Given ${\overset{-}{\alpha }}_{\text{rand}}$ and ${\overset{-}{\beta }}_{\text{rand}}$, the random information baseline *I*_rand_ can be calculated according to Equation (4). We generally selected parameters in such a way that *I*_rand_ is close to zero. However, for a number of parameter sweeps exploring the behavior of the BrainScaleS-ESS this condition is violated. We indicate whenever this is the case.

**Figure 3:**
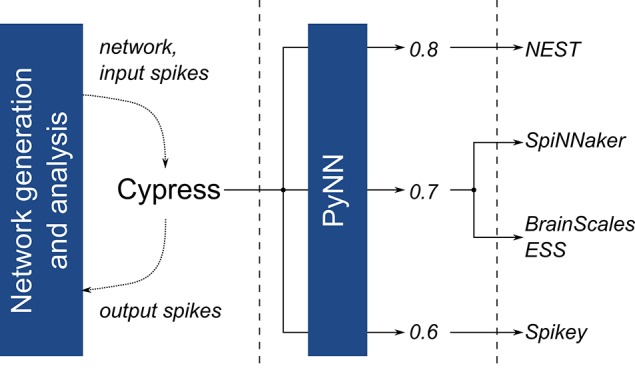
Overview of the software stack used to execute the benchmark experiments. For all platforms, the same network description and input data is passed to the Cypress C++ library, which controls the various target platforms using PyNN and platform- and API-version dependent wrapper code.

All platforms are accessed through our self-developed Cypress library, which acts as a C++ wrapper and compatibility layer for the PyNN spiking neural network description language (Davison et al., 2009). It allows to test the platforms, including their entire hard- and software stack, as black-boxes, which receive a network description and, once execution is complete, return recorded spike train data (Figure 3). Both Cypress and our benchmarking software are available online.^6^

## 3. Experiments and results

In this section we describe three independent benchmark experiments. In the first experiment we concentrate on benchmark sets which test the performance of the target platforms in scenarios with high workload, complemented by a series of follow-up experiments focusing on optimizing individual platform performance. In the second and third experiment we perform one and two-dimensional parameter sweeps, which test the platform at different levels of utilization and highlight differences in the neuron parameter mapping. To evaluate the efficiency of the systems, we propose an efficiency measure and present power measurements. We then give a short summary of the results.

### 3.1. Benchmark experiments for high-workload scenarios

In this experiment we analyze the performance of the target platforms for high-workload configurations. Here, high-workload refers to the usage of a maximal number of neurons per platform and maximal number of synapses per neuron. Spikey and the ESS are tested at full system/single chip neuron utilization (384 neurons for Spikey and 128 neurons consisting of 4 neuron circuits each for ESS). SpiNNaker is analyzed with two different input vector sizes and 1,600 neurons, which comfortably map onto a single chip. Experiments are executed with and without neuron populations and bursts, resulting in four modes of operation *(a)* to *(d)*. To mitigate stochastic effects, Spikey experiments are repeated ten times (with exactly the same input) and the results are averaged. This approach is not necessary for digital simulators, which are either deterministic (NEST, ESS) or do not exhibit variance across results (SpiNNaker). The sample count *N* is chosen as the sample count with the maximum amount of information stored in the memory. This value can be estimated by combining Equations (4, 5) and maximizing for *I*. The results, as well as data and neuron parameters are given in Table 2 and discussed in the following.

**Table 2:** Benchmark results on various platforms.

**Hardware**	**Data parameters**			**Results**				**Reference (NEST)**			
	***m***	***n***	***N***	***I*_n_**	**${\overset{-}{\alpha }}_{\text{n}}$**	**${\overset{-}{\beta }}_{\text{n}}$**	***t*/*N* [ms]**	***I*_n_**	**${\overset{-}{\alpha }}_{\text{n}}$**	**${\overset{-}{\beta }}_{\text{n}}$**	***t*/*N* [ms]**
**(A) SINGLE SPIKE, SINGLE NEURON (*s* = 1, ω = 1;*V*_th_ = −57 mV, *g*_leak_ = 20 nS, *w* = 10 nS)**											
ESS	112	128	735	0.933	0.008	0.029	2,526.30	0.974	−0.014	0.023	39.56
Spikey	256	384	4619	0.255	0.487	0.004	0.31	0.979	−0.014	0.020	121.22
SpiNNaker	1,600	1,600	1,136,48	0.917	−0.012	0.069	100.81	0.981	−0.014	0.018	1,106.43
SpiNNaker	10,000	1,600	7,102,99	0.948	−0.030	0.048	102.97	0.965	−0.033	0.034	2,106.28
**(B) SINGLE SPIKE, POPULATION (*s* = 1, ω = 4;*V*_th_ = −64.7 mV, *g*_leak_ = 20 nS, *w* = 1 nS)**											
ESS	28	32	54	0.738	−0.380	0.278	3,142.96	1.000	0.000	0.000	44.89
Spikey	64	96	324	0.393	0.287	0.084	2.69	1.000	0.000	0.000	113.24
SpiNNaker	400	400	7,499	1.000	0.000	0.000	102.89	1.000	0.000	0.000	1,454.17
SpiNNaker	2,500	400	46,866	0.999	0.000	0.000	106.86	1.000	0.000	0.000	3,010.11
**(C) BURST, SINGLE NEURON (*s* = 4, ω = 1;*V*_th_ = −64.7 mV, *g*_leak_ = 20 nS, *w* = 1 nS)**											
ESS	112	128	735	0.858	−0.131	0.138	2,507.62	0.981	−0.019	0.019	39.56
Spikey	256	384	4,619	0.250	0.112	0.488	0.34	0.980	−0.021	0.021	112.09
SpiNNaker	1, 600	1, 600	1,136,48	0.979	−0.021	0.021	102.40	0.981	−0.019	0.019	1,220.60
SpiNNaker	10, 000	1, 600	7,102,99	0.985	−0.015	0.015	118.32	0.982	−0.018	0.018	1,598.37
**(D) BURST, POPULATION (*s* = 4, ω = 4;*V*_th_ = −62 mV, *g*_leak_ = 89 nS, *w* = 1 nS)**											
ESS	28	32	54	0.000	−1.000	1.000	3,145.00	0.909	−0.011	0.074	45.20
Spikey^†^	64	96	324	(0.240)	(0.519)	(0.038)	(3.00)	0.936	0.002	0.046	169.78
SpiNNaker	400	400	7, 499	0.776	−0.119	0.204	103.85	0.935	0.001	0.049	1,473.98
SpiNNaker	2, 500	400	46, 866	0.980	0.001	0.015	108.46	0.976	−0.016	0.022	3,121.54

*Symbols: m input size, n output size, N number of samples, I_n_ normalized information, ${\overset{-}{\alpha }}_{\text{n}}$ normalized average false positives, ${\overset{-}{\beta }}_{\text{n}}$ normalized average false negatives, t/N simulation time per sample, s burst spike count, ω population size. For all experiments c = 4 (number of ones in the input ${\vec{x}}_{k}$) and d = 4 (number of ones in the output ${\vec{y}}_{k}$). Refer to Table 1 for neuron parameters. Darker colors indicate suboptimal results*.

† *g_leak_ is outside of the supported range for Spikey. Results are not valid and only included for reference. See text*.

#### 3.1.1. NEST

The NEST reference simulations yield near-optimal performance ratings in almost all cases. This is unsurprising since the neuron parameters are tuned in such a way, that the mathematical neuron model implemented in NEST reproduces the behavior of a theoretical McCulloch-Pitts cell, which in turn is the neuron type underpinning the theoretical model (compare Section 2.3). In practice, *I*_n_ is slightly smaller than one due to the additive spike-time noise in the input (Section 2.2).

#### 3.1.2. SpiNNaker

The SpiNNaker platform produces results close to NEST. Some larger deviations are visible in experiment *(d)*. Here, a relatively high false negative rate indicates that spikes might have been lost. Interestingly, the issue does not occur for an increased input count, pointing at a potential software problem.^7^ Another potential reason may be the limited accuracy of the numerical solver, which we analyze in Section 3.2.

#### 3.1.3. Spikey

In most cases, Spikey reaches about one quarter of the theoretically possible recallable information. An exception is experiment *(b)* with enabled neuron populations. This increases the information to *I*_n_ = 39%. Additional activation of bursts in experiment *(d)* results in a performance decrease, since the corresponding neuron parameters cannot be mapped to the hardware (compare Table 1). We were not able to find parameters which at the same time fulfill the threshold condition (Sections 2.2 and 2.3) and fall into the Spikey parameter range. In general, log files produced by the Spikey software report discarded input spikes, yet provide no quantitative information regarding lost output spikes. In setup *(a)* a warning regarding potential output spike loss is triggered (“event buffer half full”). We would expect spike loss to result in an elevated level of false negatives, however, this is not the case and we observe a high number of false positives instead, causing the warning in the first place. Thus, we conclude that spike loss is not a problem in our Spikey experiments.

#### 3.1.4. BrainScaleS-ESS

The BrainScaleS-ESS reaches *I*_n_ ≈ 94% for setup *(a)* with only a small amount of false positives and negatives. Setups *(b)* and *(c)* reach lower *I*_n_ ≈ 74% and *I*_n_ ≈ 86%, respectively, caused by an increased number of false negatives ${\overset{-}{\beta }}_{\text{n}}\approx 28\%$ and ${\overset{-}{\beta }}_{\text{n}}\approx 14\%$. These can be explained with provenance data recorded by the simulator, which tracks input spikes lost in off-wafer communication networks, as well as spike loss at the on-wafer output spike encoders. Note that experiments expanding on the following assertions are presented in Section 3.2.

Setup *(a)* results in input spike loss below 1%, which on average does not significantly influence the result. For *(b)* about 6% of all input spikes and 4% (54 spikes) of all output spikes are discarded, which roughly accounts for the number of false negatives observed. For *(c)* about 3% of all input spikes—0.5 spikes per sample—are discarded, which again corresponds to the average ${\overset{-}{\beta }}_{\text{n}}\cdot d\approx 0.55$ false negatives per sample. The additional output spike loss in *(b)* compared to *(c)* is likely caused by the population coding, which triggers several spikes in different neurons at the same time, resulting in high bandwidth requirements in the output spike encoders.

For the last setup *(d)* we observe no output spikes at all. Note that in population coding incoming spikes are packed more densely, since the input spikes are triggered at approximately the same moment in time. In conjunction with input bursts, this temporarily results in a high peak network load, causing 40% loss on the way to the wafer, which corresponds to *s* · ω · *c* · 0.4 ≈ 25 lost spikes per sample. Remember that neuron parameters were optimized in such a way that a single output spike is produced for about *s* · ω · (*c*−1)+1 = 49 input spikes. Therefore, it is highly unlikely that a sufficient number of spikes arrives at a single neuron, explaining the lack of output spikes.

### 3.2. Optimizing platform performance for high-workload scenarios

Experiments in the previous section 3.1 focused on the comparison of networks with exactly the same neuron parameters. Furthermore, common platform configurations encountered by end-users were used. Nevertheless, the above experiments may be rightfully criticized for not fully exploiting the capabilities of each specific target platforms. In the following, we investigate in how far the benchmark results can be improved when specifically tailoring the experiments to the target platform.

#### 3.2.1. SpiNNaker and NEST

As described in Section 2.4, the SpiNNaker system uses an Euler integrator with a a default time step of 1 ms to solve neuron and synapse dynamics. In contrast, our reference NEST simulations use an adaptive Runge-Kutta-Fehlberg integrator and a maximum timestep of 0.1 ms. In the following, we compare SpiNNaker results to NEST simulations using an Euler integrator with consistent 0.1 and 1 ms timesteps on both platforms.

Results for these experiments are shown in Table 3. For a 0.1 ms timestep there is virtually no difference between results for the NEST and SpiNNaker simulations, with the SpiNNaker simulation being slightly better. For both platforms, a 1 ms timestep reduces the overall performance in most cases, although the SpiNNaker Euler integrator performs far better (with *I*_n_ between 91% and 100%) than our NEST Euler implementation, where performance is reduced tremendously to information levels as low as 52%. These results confirm that the SpiNNaker integrator is more robust than a naïve Euler integrator implementation. Furthermore, for this experiment, there are no hardware-specific bottlenecks causing a reduction in the network performance. However, it should be noted that our single-threaded NEST simulations are about ten times faster for a network of 1600 neurons filling a single SpiNNaker chip.

**Table 3:** Benchmark results on SpiNNaker compared to a naïve Euler-integrator implemented in NEST with different timesteps for the integrator.

**Time step**	**Data Parameters**			**SpiNNaker**				**NEST-Euler**			
***Δt* [ms]**	***m***	***n***	***N***	***I*_n_**	**${\overset{-}{\alpha }}_{\text{n}}$**	**${\overset{-}{\beta }}_{\text{n}}$**	***t*/*N* [ms]**	***I*_n_**	**${\overset{-}{\alpha }}_{\text{n}}$**	**${\overset{-}{\beta }}_{\text{n}}$**	***t*/*N* [ms]**
**(A) SINGLE SPIKE, SINGLE NEURON**											
0.1	1,600	1,600	113,648	0.973	−0.013	0.024	1,003.19	0.981	−0.012	0.018	121.80
1.0	1,600	1,600	113,648	0.917	−0.012	0.069	100.81	0.557	0.188	0.001	11.24
**(B) SINGLE SPIKE, POPULATION**											
0.1	400	400	7,499	0.998	0.000	0.001	1,008.23	0.985	0.005	0.000	104.70
1.0	400	400	7,499	1.000	0.000	0.000	102.89	0.529	0.220	0.000	10.84
**(C) BURST, SINGLE NEURON**											
0.1	400	400	7,499	0.978	−0.022	0.022	1,009.84	0.958	−0.015	0.015	109.84
1.0	400	400	7,499	0.979	−0.021	0.021	102.40	0.933	0.022	0.002	11.34
**(D) BURST, POPULATION**											
0.1	400	400	7,499	0.926	0.001	0.057	1,013.192	0.929	0.004	0.047	113.68
1.0	400	400	7,499	0.776	−0.119	0.204	103.85	0.764	0.095	0.006	12.72

*Darker colors indicate suboptimal results*.

#### 3.2.2. Spikey

As described in Section 2.4, we measured an excitatory time constant of τ_exc_ = 2 ms on our Spikey system and optimized the neuron parameters for this value accordingly. To verify that this assumption does not decrease performance, we evaluated a second set of benchmarks with τ_exc_ = 5 ms, which is the internal target value of the calibration routine for the Spikey system.

In contrast to the previous experiments, we were not able to find optimal parameters in this regime of the parameter space for all scenarios, which is reflected in the reduced *I*_n_ for the NEST reference simulation in scenario *(d)*. Results for NEST and Spikey with τ_exc_ = 5 ms are both given in Table 4. In comparison to the previous experiments, Spikey performance is significantly better in experiments *(a)* and *(b)*. Especially in the latter setting performance increased from roughly 40% to 65%. Interestingly, and exactly as before, performance is worse in experiment *(c)* with an increased false negative count, although the same neuron parameters have been used. The same phenomenon can be observed when comparing *(b)* and *(d)*, suggesting that the switch to burst coding triggers this problem. Analogous to Section 3.1, spike loss has not explicitly been reported for these experiments. Therefore, we assume that deviations are caused by imprecise neuron parameter translation and noise.

**Table 4:** Benchmark results on Spikey with assumed τ_exc_ = 5 ms.

**Data Parameters**			**Spikey**				**Reference (NEST)**			
***m***	***n***	***N***	***I*_n_**	**${\overset{-}{\alpha }}_{\text{n}}$**	**${\overset{-}{\beta }}_{\text{n}}$**	***t*/*N* [ms]**	***I*_n_**	**${\overset{-}{\alpha }}_{\text{n}}$**	**${\overset{-}{\beta }}_{\text{n}}$**	***t*/*N* [ms]**
**(A) SINGLE SPIKE, SINGLE NEURON**										
256	384	4,619	0.469	0.201	0.069	0.23	0.990	−0.008	0.010	122.84
**(B) SINGLE SPIKE, POPULATION**										
64	96	324	0.643	0.117	0.066	2.52	0.909	0.037	0.000	117.30
**(C) BURST, SINGLE NEURON**										
256	384	4,619	0.336	−0.318	0.608	0.26	0.995	−0.001	0.004	117.86
**(D) BURST, POPULATION**										
64	96	324	0.291	−0.736	0.737	2.67	1.000	0.000	0.000	124.23

Refer to the Spikey^*^ neuron parameters in Table 1. Darker colors indicate suboptimal results.

#### 3.2.3. BrainScaleS-ESS

As elaborated in the previous section, provenance data show that a part of the false negatives observed in our benchmark are related to loss of input spikes on their way to the virtual wafer. The standard mapping algorithm inserts spikes from external neurons into HICANNs that are physically close to their target neurons (Jeltsch, 2014). This results in all input spike trains being routed via few HICANNs with limited input bandwidth (Thanasoulis et al., 2014). This bandwidth is most likely to be exceeded in scenarios *(b)*-*(d)*, where spike bursts or neuron populations produce relatively high peak spike rates. To address this problem, the current software allows to consider the firing rate of external neurons and the input bandwidth for the distribution of inputs to HICANNs, providing a larger number of physical paths for the spike data. We executed the same experiments from Table 2 with this option to test whether this enhances performance.

The results in Table 5 show minor improvements in scenarios *(a)* and *(b)*. In setup *(b)* false negatives are mainly caused by output spike loss, which is unchanged compared to previous experiments. Relatively large improvements are visible for setups *(c)* and *(d)*. In setup *(c)* the input spike loss is reduced to below 1%, yielding results similar to *(a)*. In setup *(d)* input spike loss is reduced to about 2%, with an additional output spike loss of about 7%. However, these spike loss figures do not fully account for the still high average false negative count of ${\overset{-}{\beta }}_{\text{n}}=0.43$. As indicated by the relatively low performance of the reference simulation, this experimental setup is particularly sensitive with respect to noise in neuron and synapse parameters as well as spike timing. This suggests that neuron and synapse parameter discretization along with potential spike time jitter in the ESS may be responsible for the remaining error.

**Table 5:** Simulation results for the BrainScaleS-ESS using an increased bandwidth for spike insertion.

**Data Parameters**			**ESS**				**NEST**			
***m***	***n***	***N***	***I*_n_**	**${\overset{-}{\alpha }}_{\text{n}}$**	**${\overset{-}{\beta }}_{\text{n}}$**	***t*/*N* [ms]**	***I*_n_**	**${\overset{-}{\alpha }}_{\text{n}}$**	**${\overset{-}{\beta }}_{\text{n}}$**	***t*/*N* [ms]**
**(A) SINGLE SPIKE, SINGLE NEURON**										
112	128	735	0.936	0.008	0.026	3,219.65	0.974	−0.014	0.023	39.56
**(B) SINGLE SPIKE, POPULATION**										
28	32	54	0.782	−0.275	0.222	4,480.50	1.000	0.000	0.000	44.89
**(C) BURST, SINGLE NEURON**										
112	128	735	0.925	−0.052	0.069	3,575.77	0.981	−0.019	0.019	39.56
**(D) BURST, POPULATION**										
28	32	64	0.564	−0.421	0.431	11,277.93	0.909	−0.011	0.074	45.20

*Neuron parameters in Table 1. Darker colors indicate suboptimal results*.

### 3.3. One-dimensional data parameter sweeps

Here we conduct one-dimensional sweeps of the data parameters *m, n, c, d* (Section 2.1). See Figure 1 for graphs of the normalized information *I*_n_, and the supplemental material for ${\overset{-}{\alpha }}_{\text{n}}$ and ${\overset{-}{\beta }}_{\text{n}}$.

For a constant sample count *N*, the parameter *m* controls the number of possible synapses per neuron, whereas *n* controls the number of neurons. In the optimal case, the normalized information measure would stay at 100% regardless of the values for *m* and *n*. This behavior is observed for the NEST, SpiNNaker, and BrainScaleS-ESS simulations. As in previous benchmarks, the performance of Spikey ranges from 20% to 65% of the theoretical performance, with an almost linear increase in information capacity for both sweeps over *m* and *n*. A discontinuity is visible at *n* = 192, the number of neurons in a single Spikey block.^8^ A possible explanation for the increase of performance with growing *m*, *n* is analog crosstalk. As the network is scaled up, *P*((*M*)_*ij*_ = 1) decreases, reducing the number of neurons which spike or are driven to high subthreshold regimes without spiking. Since more neurons are in low membrane potential states, fluctuations caused by activity in neighboring neuron and synapse circuits is less likely to trigger false-positive spikes. This is visible as a decrease in false positives ${\overset{-}{\alpha }}_{\text{n}}$.

The sweep over the parameter *c*, the number of ones in the input patterns, should cause a peak in *I*_n_ at *c* = 4, since the neuron parameters have been tuned to this value (Section 2.2). Here, NEST, ESS, SpiNNaker, and Spikey^*^ (referring to the second set of parameters, Section 3.2) behave as expected. A similar peak is visible for Spikey with the first set of parameters at a lower number of bits, demonstrating that the chosen parameters would be better suited for simulating a BiNAM with *c* = 3 and highlighting the parameter mismatch. In the case of the the ESS experiment—which uses smaller *m* and *n* to reduce simulation times—the random information baseline *I*_rand_ is significantly greater than zero for large *c*. As spike losses in the ESS suppress a large number of false positives predicted by the theoretical model, the normalized information capacity *I*_n_ surpasses its theoretical maximum value of one. As discussed in Section 2.5, these results highlight that *I*_rand_ ≈ 0 must be ensured when performing benchmark experiments.

Theoretically, variation of the number of active bits in the output pattern *d* should not have any effect on *I*_n_, as is the case for the NEST and SpiNNaker simulations. As before, both show normalized information values near *I*_n_ = 100%. For Spikey, the measure decreases along with a higher overall network activity, pointing at the aforementioned crosstalk problems, which is further supported by the increasing number of false positives (see Supplemental Material). The ESS behaves similarly as in the last experiment. For larger values of *d*, the performance decreases as spikes are lost on the way to and from the neurons. However, as *d* increases even further, the random information baseline *I*_rand_ becomes much greater than zero. Correspondingly, the loss of a significant amount of false positives seemingly causes an increase in performance.

### 3.4. Two-dimensional neuron parameter sweep

**Figure 4:**
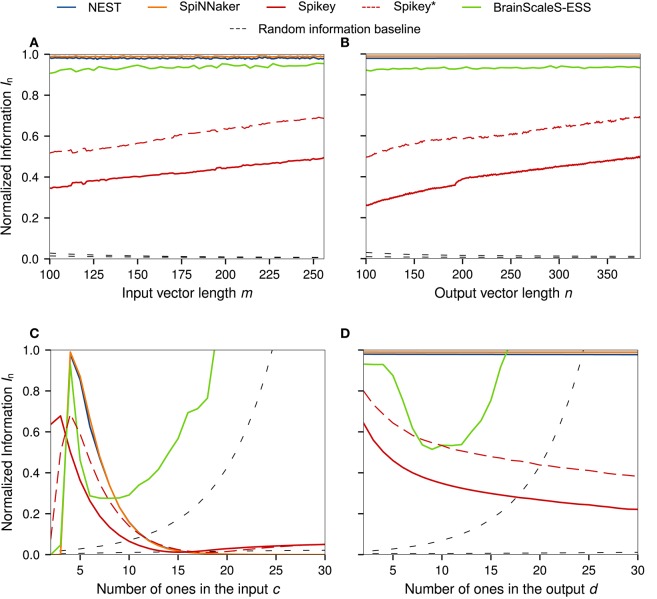
One dimensional sweeps over the input and output **(A,B)** vector size *m, n*, and the number of ones in the input and output **(C,D)** patterns *c, d*. Standard network size of all sweeps is *n* = 256, *m* = 384, *c* = 4, *d* = 4, and *N* = 1, 000. The results for Spikey^*^ refer to an alternative parameter set generated under the assumption that τ_exc_ = 5 ms. All simulations were averaged over five runs with different random seeds for the data generation. For the ESS we only evaluated single simulations with standard parameters of *m* = 112, *n* = 128, and *N* = 735 and reduced resolution to decrease simulation times. The normalized random information baseline is calculated according to Equation (12) with *c*^th^ = 4. Of the two graphs in each subfigure, the one reaching larger *I*_n_ is based on the reduced matrix size in the ESS experiment.

**Figure 5:**
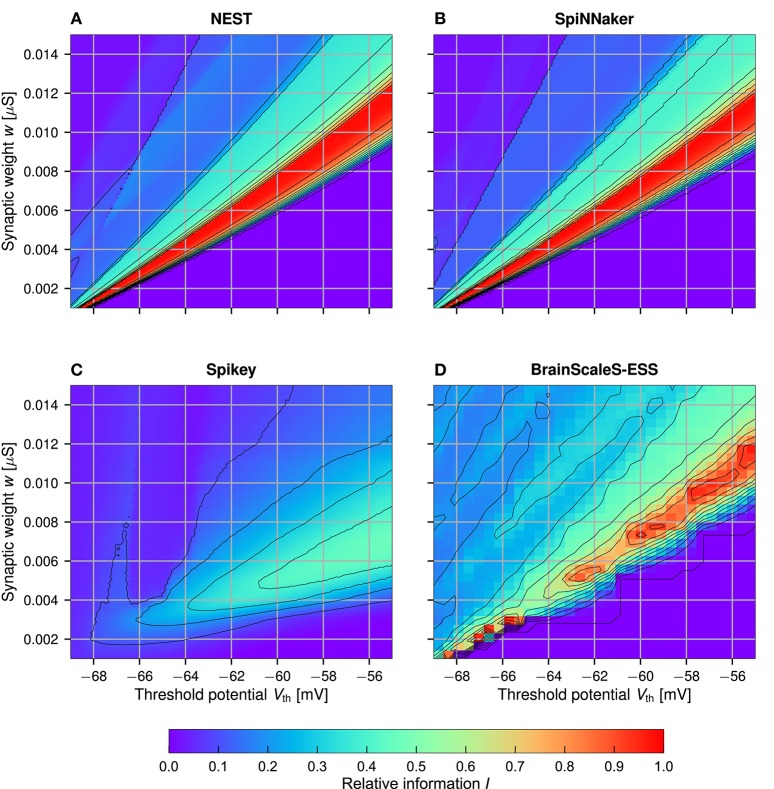
Sweep over the two neuron parameters *V*_th_ and *w* for NEST **(A)**, SpiNNaker **(B)**, Spikey **(C)**, and BrainScaleS-ESS **(D)**. Neuron parameters are set to *n* = 256, *m* = 384, *c* = 4, *d* = 21, and *N* = 1, 000 (optimum), for the ESS these are changed to *n* = 112, *m* = 128, *c* = 4, *d* = 4, and *N* = 735 (again at optimal storage capacity). Shown is the normalized information *I*_n_. For Spikey results are averaged over five runs **(C)**. The value of *d* was set to a higher number to reduce the number of samples and with it the simulation time. Note that the sweep resolution is small for the ESS **(D)**, resulting in aliasing artifacts.

As already mentioned, a possible explanation for the inferior results for Spikey is an incorrect translation of the neuron model parameters to the analog hardware parameters. In this experiment we explore the parameter space with a two dimensional sweep over the threshold potential *V*_th_ and the synaptic weight *w*. These parameters are in an easily understandable relationship: given a parameter set for which the memory works perfectly, an increase in the threshold potential *V*_th_ requires larger *w* for the neuron to produce output spikes. Results for *I*_n_ are depicted in Figure 5, results for ${\overset{-}{\alpha }}_{\text{n}}$ and ${\overset{-}{\beta }}_{\text{n}}$ can be found in the supplemental material. Note that *I*_rand_ is significantly larger than zero in the ESS experiments (cf. Figure 4). Correspondingly, the reported values for *I*_n_ should not be understood as an absolute performance measure, but solely as a means to characterize the ESS parameter space.

Both NEST and SpiNNaker reach the perfect normalized information *I*_n_ = 1 in a large region of the parameter space. Here, an increase in *V*_th_ must be met with an almost linear increase in *w*. Furthermore, for larger *V*_th_ the range of synaptic weights *w* which produce a perfectly working memory becomes larger, allowing for a more robust memory with respect to parameter noise. Slight differences between NEST and SpiNNaker, as well as the slightly noisier results in SpiNNaker can be attributed to the lower precision of the numerical Euler integrator (Section 2.4). The BrainScaleS-ESS can reproduce the general parameter dependencies, while slight deviations are caused by limited resolution of neuron parameters and limited network bandwidth in areas with relatively large weights and small threshold (as discussed above). Results for Spikey differ significantly from those in NEST. No set of parameters surpasses *I*_n_ = 60%, and the region with passable results is broader, smoother, and shifted toward higher threshold potentials and smaller weights (compared to NEST), indicating a sub-optimal translation of neuron parameters. We also evaluated a second set of parameters on the Spikey system, as with our previous experiments. However, there are no significant differences to the results shown here, although *g*_leak_ was significantly changed from 20 nS to 39 nS. For reference, the results for the second sweep are included in the supplemental material.

### 3.5. Power-efficiency

In this section we compare the power consumption of the Spikey and SpiNNaker neuromorphic hardware systems. We did not analyze the ESS, since the power consumption of the simulation is unrelated to the consumption of the actual hardware system. For a more comprehensive analysis of the setup times of networks in PyNN refer to (Diamond et al., 2015). Note that we deliberately decided not to include common power measures such as energy per synaptic event, as individual neurons are either in or relaxing toward their resting state most of the time, which would heavily bias a per-spike power measure toward the static energy consumption of the system.

Power measurements have been conducted directly in the 5 V supply line during network simulation and platform-specific setup for both hardware systems. For the four chip SpiNNaker board we measured 220 mA at 5.09 V during simulation^9^, resulting in a power-consumption of (1.12 ± 0.05)W. Spikey consumes 1.11 A at 5.28 V, equaling (5.84 ± 0.06)W. Even though power consumption of a NEST simulation highly depends on the computer hardware being used, we at least try to provide a rough estimate for the laptop computer with Intel Core i7-4710MQ processor used for the presented benchmarks. The power consumption of the system idling is approximately 11 W, and approximately 29 W while NEST simulations are running.^10^ We calculate efficiency based on the difference of 18 W to exclude static power consumption of peripheral devices such as the display from our calculations.

As a measure for energy efficiency *E*_eff_, we propose the number of samples that can be recalled per joule, scaled by the normalized information *I*_n_

(13) $$\begin{array}{l} E_{\text{eff}}=\frac{I_{\text{n}}\cdot N}{E}=\frac{I_{\text{n}}\cdot N}{P\cdot t}\cdot \end{array}$$

Table 6 shows the energy requirements of both hardware platforms for a selected set of experiments from Section 3.1. Spikey is about 15 times more efficient than SpiNNaker, even though it only allows to retrieve a quarter of the theoretically expected information. Clearly, the reason for this discrepancy is the speed-up of the physical system by a factor of 10^4^ compared to biological realtime, while the SpiNNaker system is set to realtime. Nevertheless, these values should be interpreted with care. We used approximately one quarter of the resources available on a four-node SpiNNaker board, but measured its total energy consumption. In addition, the sample presentation time was set to 100 ms, preventing interference between the sample recalls, although values up to three times smaller are realizable on SpiNNaker, greatly improving the energy efficiency. On Spikey simulation times are already extremely short (e.g., for 4,619 samples simulation itself takes 46.19 ms, while the total execution time is 1.4 s). Hence, the energy consumption on this platform is dominated by setup and teardown processes executed on the host computer, e.g., neuron mapping. These are also included in SpiNNaker and NEST simulations, but require a smaller relative timespan. This is the main reason for the comparatively small differences in efficiency: a speed-up factor of 10^4^ at a quarter of the normalized information and a factor of five in power consumption would amount to much larger differences in theory. The NEST simulations, when executed on a system with an Intel Core i7-4710MQ processor, aiming at accuracy instead of efficiency, are far off concerning energy consumption. However, while our naïve implementation of the Euler-integrator in NEST—running on a more modern chip fabricated in a smaller semiconductor technology—consumes more energy than SpiNNaker, energy efficiency is in the same order of magnitude. Further potential for improvement exists, since our NEST-Euler implementation is not as rigorously optimized as the SpiNNaker implementation. To summarize, the analog Spikey system is considerably more efficient than both SpiNNaker and simulations on standard computer hardware. Tests with far larger networks are required to asses the true potential of SpiNNaker.

**Table 6:** Energy consumption and efficiency of SpiNNaker (smaller benchmarks) and Spikey according to Equation (13).

**Platform**	**Hardware system**		**NEST**		**NEST-Euler**	
	***E*/*N* [mJ]**	***E*_eff_ [1/J]**	***E*/*N* [mJ]**	***E*_eff_ [1/J]**	***E*/*N* [mJ]**	***E*_eff_ [1/J]**
**(A) SINGLE SPIKE, SINGLE NEURON**						
SpiNNaker	112.9 ± 5.04	8.1 ± 0.4	21,000	0.05	200	2.75
Spikey	1.8 ± 0.01	141.5 ± 1.5	23,000	0.42	38	14.66
**(B) BURST, SINGLE NEURON**						
SpiNNaker	114.7 ± 5.12	8.5 ± 0.4	23,000	0.04	200	4.57
Spikey	2.0 ± 0.02	125.5 ± 1.2	23,000	0.42	38	24.40

*Experiment parameters listed in Table 2. Power consumption for NEST and NEST-Euler simulations are only a rough estimate (see text). NEST-Euler refers to the trivial Euler integrator with 1 ms timestep discussed in Section 3.2*.

### 3.6. Summary

In all experiments presented above, only minuscule deviations between SpiNNaker results and NEST simulations are visible, whereas Spikey and the BrainScaleS-ESS deviate from the reference behavior. Neuron parameter optimization specifically for the Spikey analog neuromorphic hardware system yields a normalized information value of up to *I*_n_ ≈ 64%. The presented data parameter sweeps point at interference between neighboring neuron circuits as a possible source for this discrepancy, as well as not perfectly calibrated neuron parameters. For the BrainScaleS-ESS, its internal book keeping of spikes lost during simulation allows to interpret its behavior in terms of limited available bandwidth on the on-wafer communication network. The maximal information value achieved was about *I*_n_ ≈ 94%. Due to extremely long emulation runtimes, which render large-scale experiments rather cumbersome, experiments with the actual hardware are necessary to reliably characterize the performance of the BrainScaleS system, especially since the actual BrainScaleS hardware may suffer from similar analog interference and neuron parameter mismatch problems similar to those observed in Spikey. Our proposed energy efficiency measure suggests that Spikey is at least one order of magnitude more efficient than SpiNNaker. More detailed experiments are required to support this claim.

## 4. Discussion

We introduced the binary neural associative memory (BiNAM) as a benchmark for neuromorphic hardware systems and conducted tests on SpiNNaker, Spikey, and the BrainScaleS-ESS platforms with the software simulator NEST as a reference. We successfully demonstrated that the regular, well-interpretable, and arbitrarily scalable structure of the benchmark implicitly tests the realization of the mathematical neuron model. In conjunction with recorded provenance data the benchmark can be used to obtain information about possible networking or buffer bottlenecks, deficiencies in the neuron parameter mapping process, and analog signal crosstalk.

Furthermore, we proposed a simple efficiency measure, which takes time, power consumption, and quality of information retrieval into account. We concluded that Spikey, even though inferior in quality, is at least one order of magnitude more efficient than SpiNNaker. We expect, that the Spikey system will be even more energy efficient for longer runtimes, as in our experiments the energy consumption was dominated by setup and teardown processes. However, the SpiNNaker system is undoubtedly more energy efficient than a workstation running NEST, yet reaches a similar simulation quality. Significantly larger networks or a higher workload are necessary to reach the limits of SpiNNaker.

Our experiments with a modified NEST with Euler integrator suggest that—at least for small networks—mobile consumer computer hardware reaches similar magnitudes of efficiency as SpiNNaker. However, for large networks, it is to be expected that the asynchronous computing architecture underlying SpiNNaker will be significantly more efficient as soon as the network spans a large number of chips. Conversely, our experiments suggest that a faster integrator such as the optimized 32-bit fixed-point Euler integrator implemented in SpiNNaker (Rast et al., 2010) may cause a significant speed-up for NEST simulations, without significantly reducing the accuracy of the simulator in some cases.

With respect to its suitability as a benchmark, the BiNAM has several clear advantages over other possible associative memory networks, such as Hopfield attractor networks (Hopfield, 1982). Not only is it non-trival to implement spiking attractor networks (e.g., with respect to neuron parameter selection), it is similarly hard to characterize their time-dynamics on a single-spike level under the influence of noise. This unnecessarily complicates reasoning about potential sources of errors in the underlying hardware, which we think is an important property of low-level benchmarks. In contrast, it is trivial to reason about the network behavior on an individual spike level in the context of the BiNAM. Furthermore, the evaluation of attractor network outputs is only possible as soon as the network has settled to its final state, making evaluation potentially slow, whereas the feed-forward BiNAM architecture allows to produce outputs at a very high rate. Even when considering other potential neural networks that could be used as a hardware benchmark, the BiNAM might be the simplest—and thus most assessable—neural network which can be directly translated to a spiking substrate while being a functional model of an important building block of biological brains.

Of course, it should be kept in mind that our benchmark cannot judge the suitability of the platform for bio-inspired models which are intrinsically capable of functioning even with diverse noise sources and stochastic neurons. Therefore, our conclusion from the presented data is that applications which need pre-defined and well behaving neurons, should definitely run on SpiNNaker. If the simulation needs the speed-up of Spikey, or the HICANN hardware system, we recommend tuning the behavior of the neurons with the hardware in the loop, to accommodate for the discrepancies between the physical model system and the mathematical neuron model (see for example Schmuker et al. 2014; Schmitt et al. 2017).

This necessity for parameter tuning with respect to the hardware the network is running on, is demonstrated by our Spikey experiments. Some of the neuron parameters opaquely depend on a variety of hardware parameters and may thus not be controllable by end users. Even an educated guess for one of these parameters might have been derived under circumstances which are not representative of the final network.

To implement such a hardware-in-the-loop parameter selection for the BiNAM, the optimization technique presented in Section 2.3 can be easily extended in such a way that the neuron simulations are not executed as numerical simulations, but directly run on the target system. A challenge that needs to be overcome is the high setup time for individual experimental trials on neuromorphic hardware, which quickly dominates the total runtime during batch processing. Alternatively, it is possible to multiplex several trials into a single experiment run, yet care must be taken to sample across all available physical neurons. However, and as already pointed out in Diamond et al. (2015), we think that the hardware developers should invest in reducing setup times—caused by both mapping processes on the host computer and the actual data transfer to the machine—and thus facilitate the execution of many short experiments.

To counter the fact that network connectivity itself may influence the neuron parameters (such as the synaptic weight *w* influencing the synaptic time constant τ_exc_ on Spikey, Brüderle 2009), it would be interesting to optimize the neuron parameters along with the entire network, e.g., by directly targeting the benchmark indicator *I*_n_ in the optimization process. However, this mandates a high throughput of individual experiments, which—as mentioned above—is infeasible with the current software stack provided by the hardware developers. A highly interesting direction for future research would be to execute the full feedback-driven optimization loop directly on the hardware, yet this requires thorough knowledge of the hardware system in question. For example, a future revision of the HICANN chip will include a plasticity processor intended for biologically plausible learning rules, which allows to update neuron and synapse parameters while the network is simulated (Friedmann et al., 2017).

The importance of detailed provenance data is highlighted in our BrainScaleS-ESS experiments, where we were able to significantly improve the performance of the system by analyzing both benchmark and provenance data, followed by subsequent adaptation of the experiment setup. To simplify this process for future end-users, we urge the hardware developers to provide tools which facilitate the analysis of the provenance data.

Future variants of our BiNAM benchmark could include additional recurrent connections, as proposed in one of the original BiNAM publications (Palm, 1980). This would allow to test the hardware under higher load conditions while improving the maximum memory capacity. Another promising benchmark could be an implementation of the Spike Counter Model (Knoblauch, 2003), an extension of a spiking BiNAM with additional auto-associative recurrent and inhibitory neurons acting as clean-up memories suppressing false-positives in the output. Of course, these alterations introduce additional complexity which—as discussed above—might not be desirable for low-level benchmarks.

## Author contributions

CJ ported the existing proof of concept implementation of the benchmark developed by AS to our software framework Cypress, improved on the initial ideas and performed the energy measurement experiments. Both AS and CJ contributed equally to the writing of this paper. UR initiated and supervised this work. MT and UR contributed with corrections and comments.

### Conflict of interest statement

The authors declare that the research was conducted in the absence of any commercial or financial relationships that could be construed as a potential conflict of interest.

## Footnotes

^1^A graphical tool for neuron and synapse parameter space exploration is provided at https://github.com/hbp-unibi/adexpsim.

^2^Source code is available at https://github.com/hbp-unibi/nest-simulator/tree/iaf_cond_exp_euler

^3^This refers to the HICANNv2, which is simulated by the ESS. The waferscale system uses HICANNv4, which features 220 synapses per neuron.

^4^The ESS software used in our experiments is dated June 7, 2017. Simulations are executed on a server maintained by the developers.

^5^The Spikey system used in our experiments has the serial number 506.

^6^https://github.com/hbp-unibi/cypress; https://github.com/hbp-unibi/cppnam

^7^There is a known software problem causing Spike loss in the used software version. A newer software release exists which has more severe problems with four node SpiNNaker boards, which is why, at time of writing, we are not able to analyze this issue.

^8^The standard mapping of logical neurons to hardware is linear on Spikey, causing the first 192 neurons to be situated on the first block of the system, whereas consecutive neurons are located on the second block. Simulations with random mapping do not show this discontinuity.

^9^All measurements ±10 mV and ±10 mA respectively.

^10^Measurements performed with the powertop utility while the laptop was powered by battery.
