# Supplementary figures and images for: Binary Associative Memories as a Benchmark for Spiking Neuromorphic Hardware
Source: Front Comput Neurosci. 2017 Aug 22;11:71. doi: 10.3389/fncom.2017.00071 (PMC5572441; doi:10.3389/fncom.2017.00071)

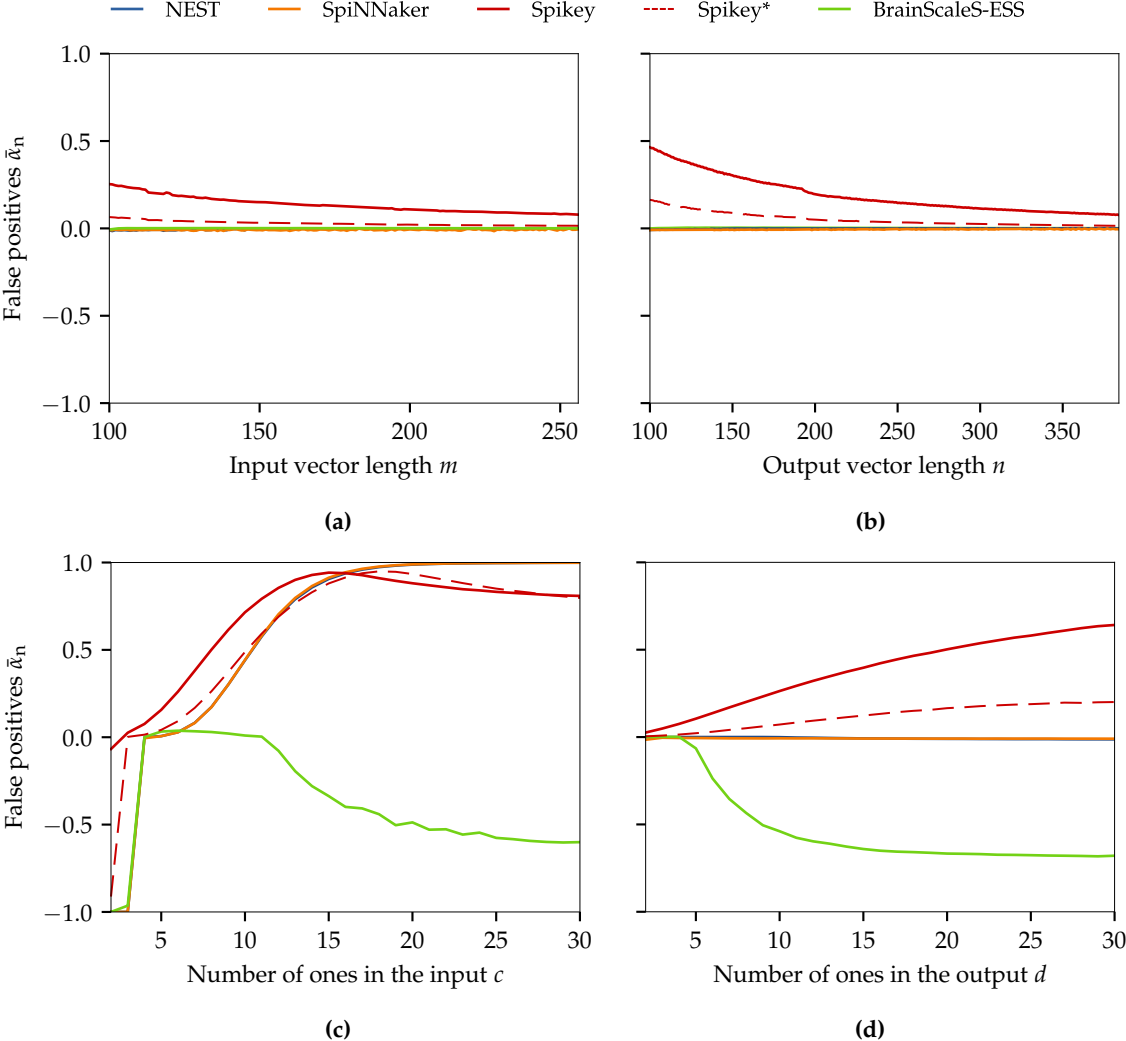

**Figure S1:** Normalized false positive count  $\bar{\alpha}_n$  for the one-dimensional parameter sweep.

Supplement: Supplementary file 1 [file Image1.pdf]

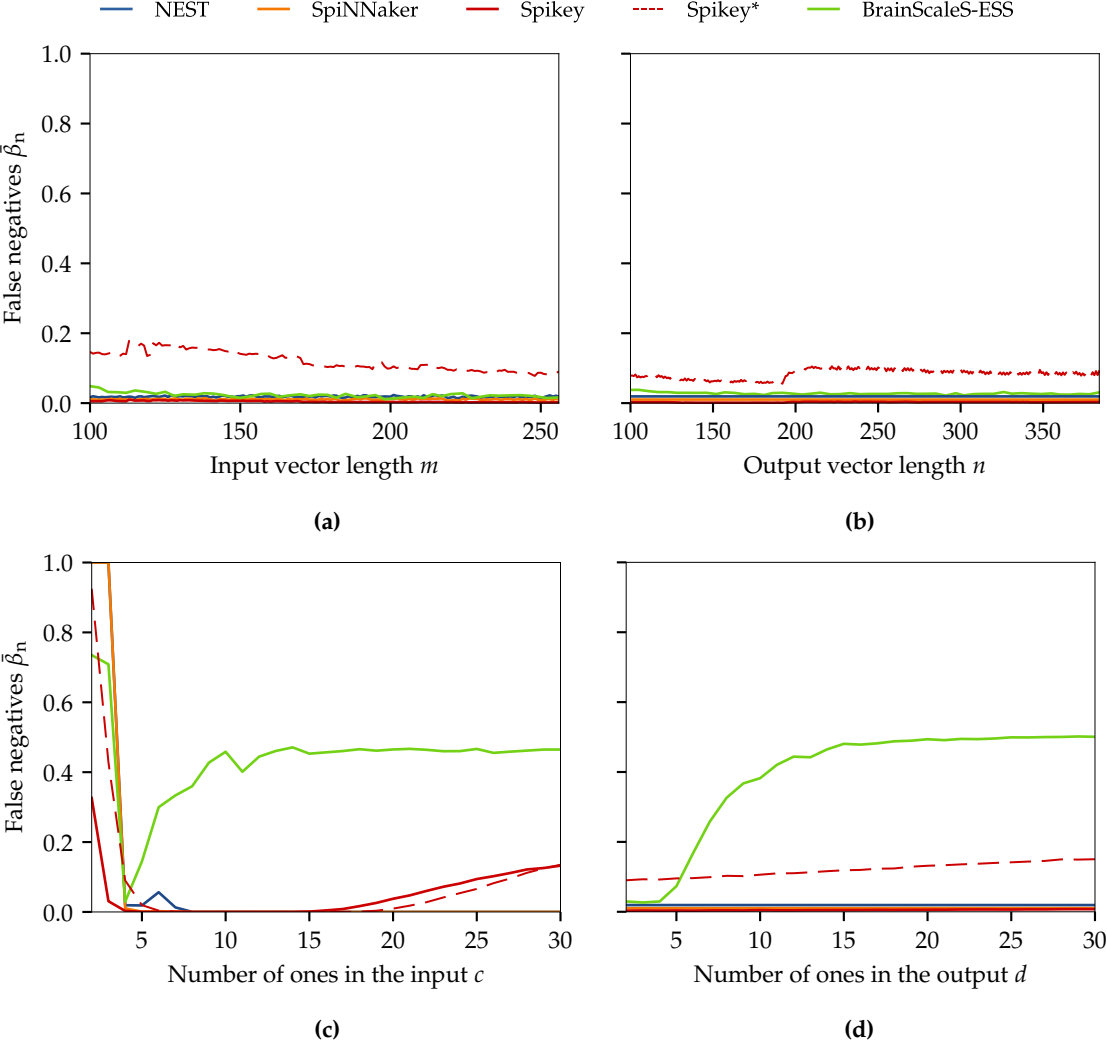

**Figure S2:** Normalized false negative count  $\bar{\beta}_n$  for the one-dimensional parameter sweep.

Supplement: Supplementary file 2 [file Image2.pdf]

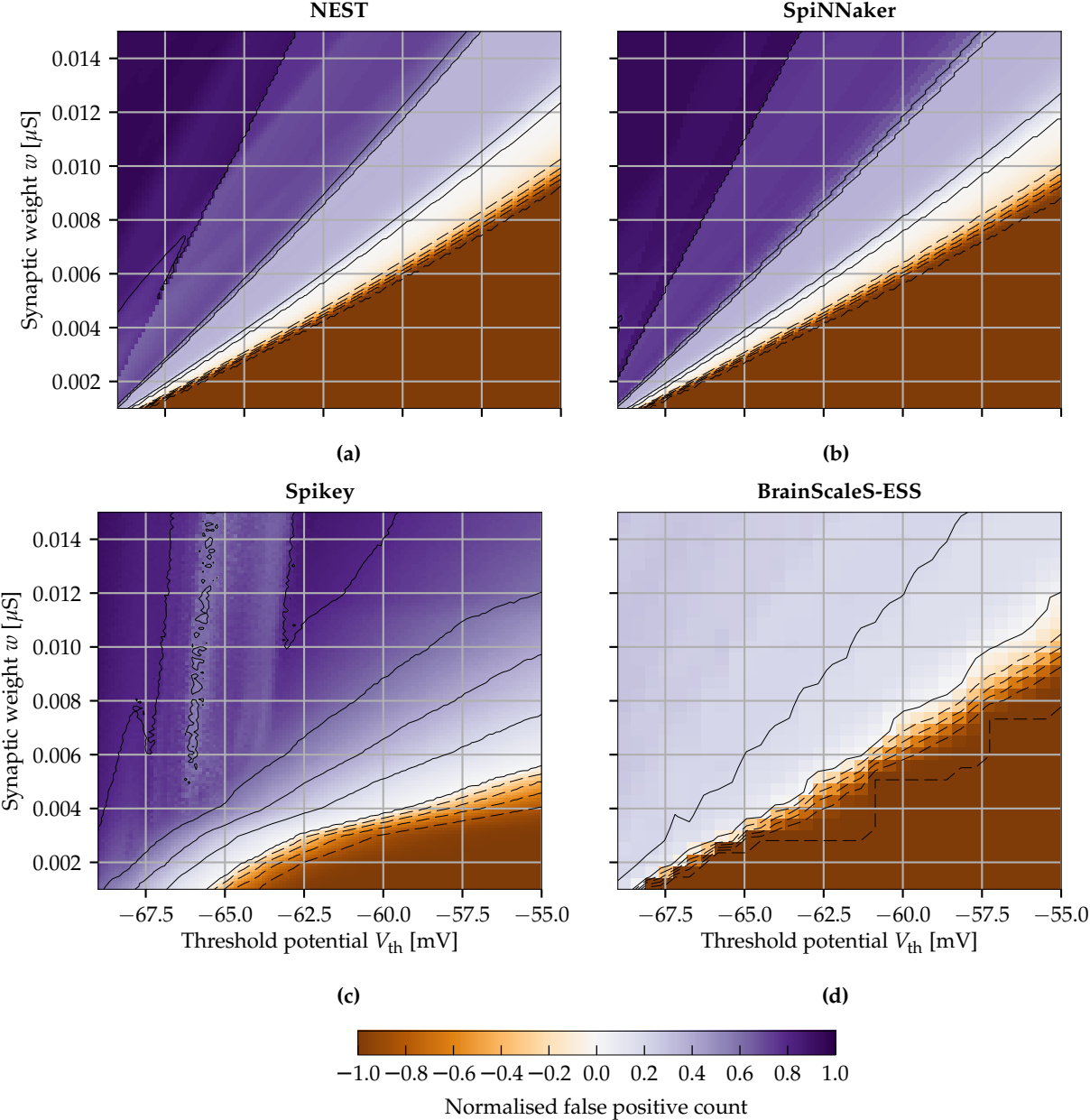

**Figure S3:** Normalized false positive count  $\bar{\alpha}_n$  for the two-dimensional parameter sweep.

Supplement: Supplementary file 3 [file Image3.pdf]

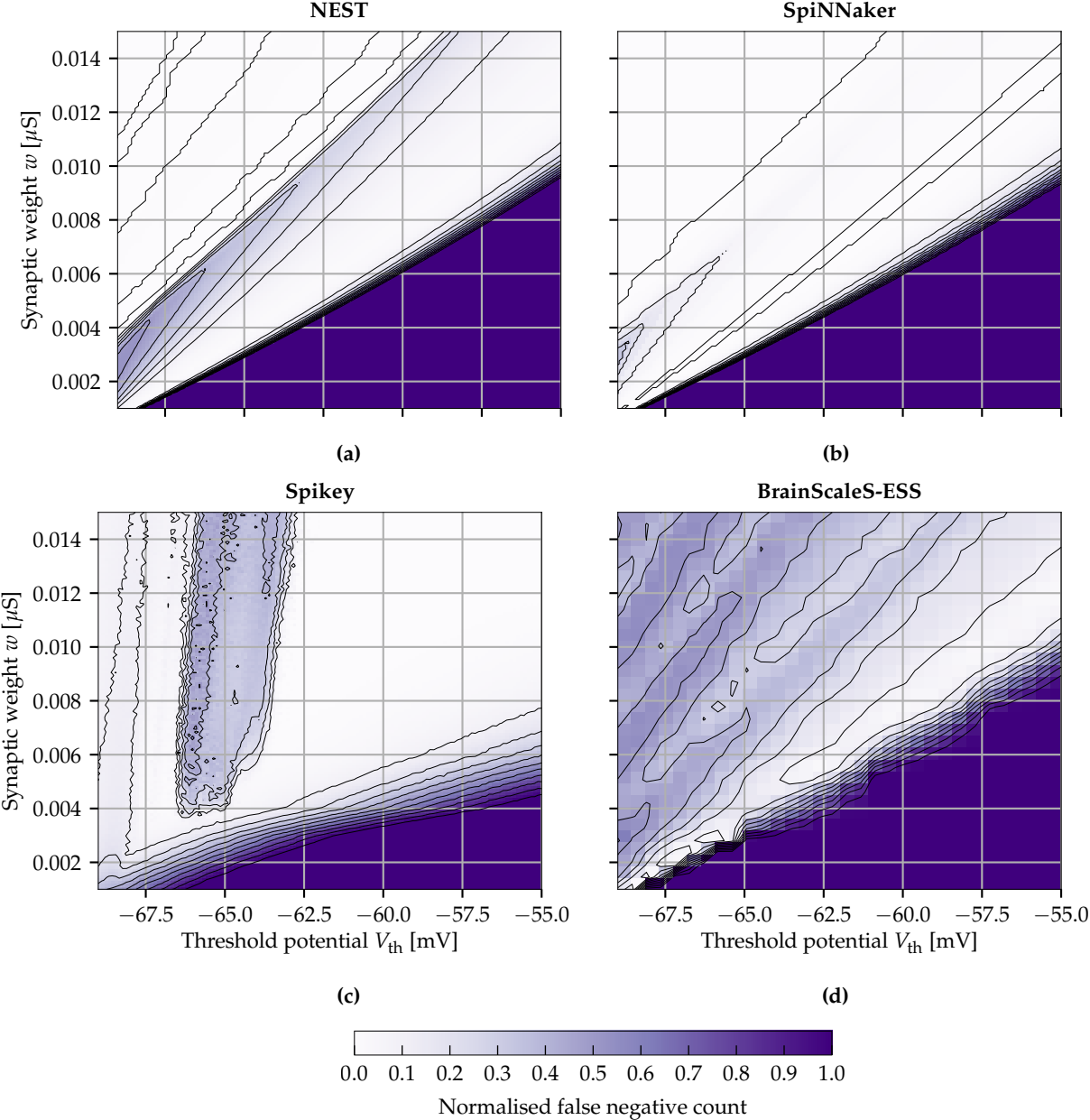

**Figure S4:** Normalized false negative count  $\bar{\beta}_n$  for the two-dimensional parameter sweep.

Supplement: Supplementary file 4 [file Image4.pdf]
