# Supplementary material for: Binary Associative Memories as a Benchmark for Spiking Neuromorphic Hardware
Source: Front Comput Neurosci. 2017 Aug 22;11:71. doi: 10.3389/fncom.2017.00071 (PMC5572441; doi:10.3389/fncom.2017.00071)
Supplement: Supplementary file 5 [file Image5.pdf]

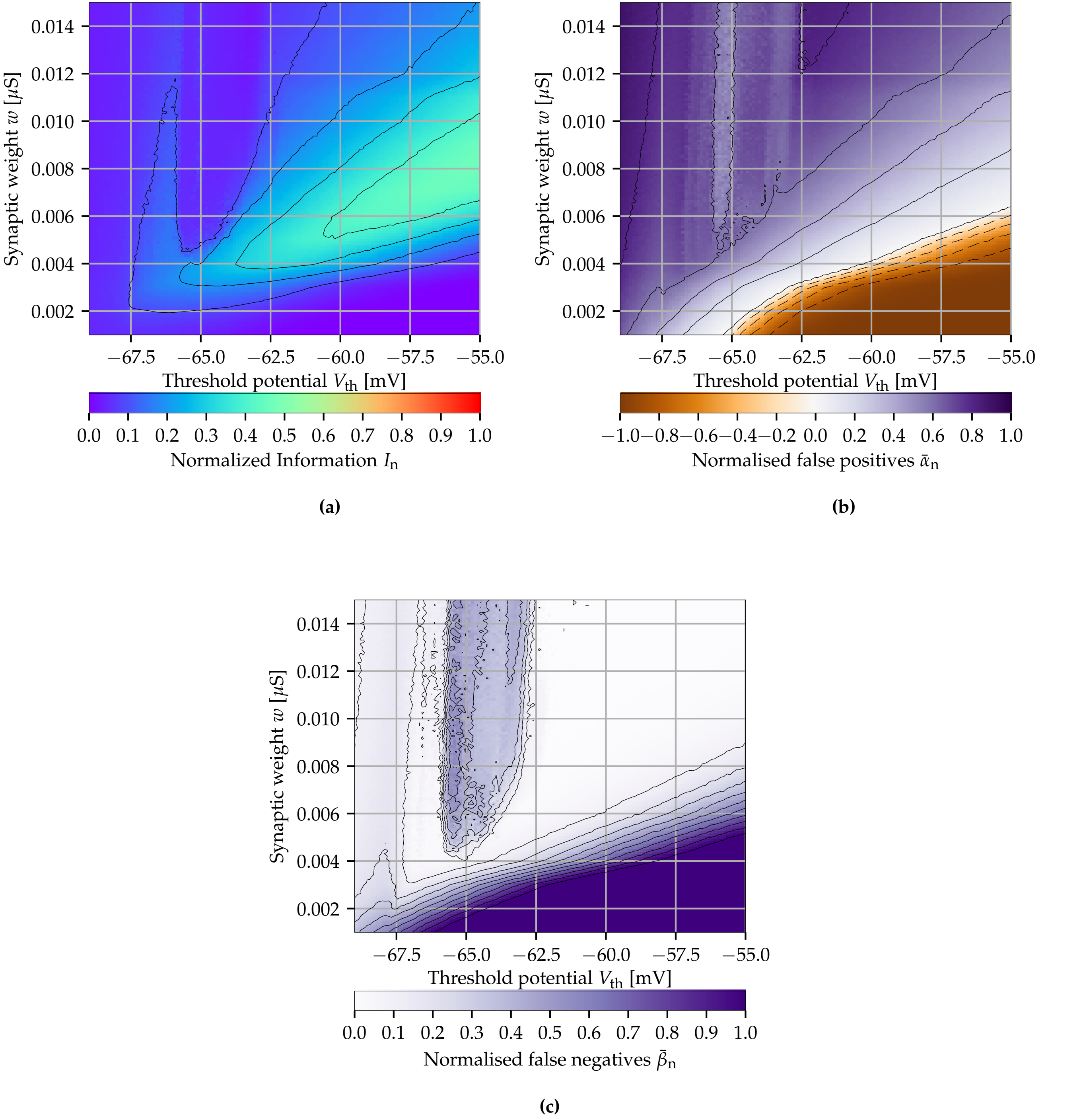

Figure S5: Normalized information I_{n}, false positive counts $\bar{\alpha}_{\mathrm{n}}$, and false negative counts $\bar{\beta}_{\mathrm{n}}$ for the two-dimensional parameter sweep on Spikey with the Spikey* parameter set.
