# Supplementary material for: Binary Associative Memories as a Benchmark for Spiking Neuromorphic Hardware
Source: Front Comput Neurosci. 2017 Aug 22;11:71. doi: 10.3389/fncom.2017.00071 (PMC5572441; doi:10.3389/fncom.2017.00071)
Supplement: Supplementary file 6 [file Image6.pdf]

Table S1: Fitted Spikey LIF parameters for three runs with varying degrees of freedom. The "initial"-column shows the initial neuron parameters from which the optimization process is started, relevant constant parameters are given in the "constants"-column. Final fitted parameters are given in the "fitted"-column along with the resulting RMSE.

**Spikey parameter fitting**								
	Init.	*Fitted*				*Constants*		
τ_{exc}	5.00	1.83	2.24	2.16	[ms]	C_{m}	0.2	[nF]
w	16.00	16.64	15.55	-	[nS]	v_{rest}	−70.00	[mV]
g_{leak}	20.00	16.37	-	-	[nS]	v_{th}	−55.00	[mV]
RMSE	526.81	56.08	68.76	68.98	[µV]			

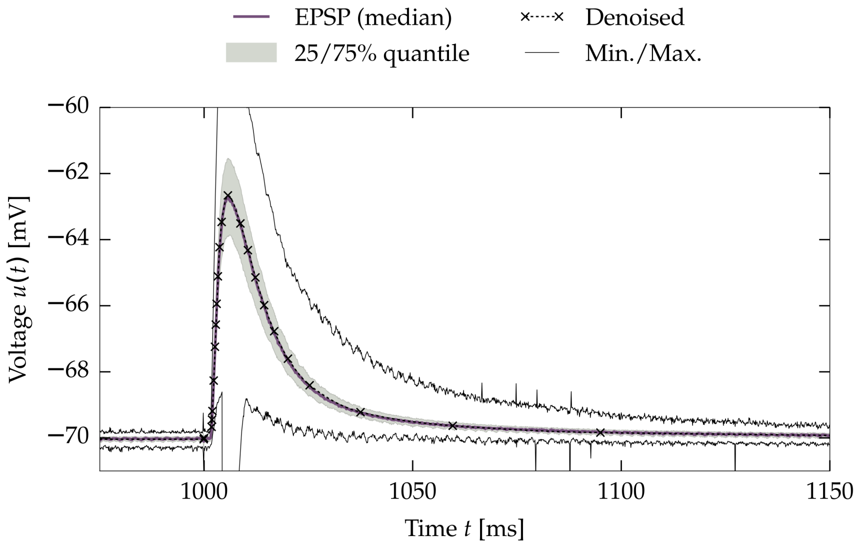

(a) Recorded and denoised EPSP traces.

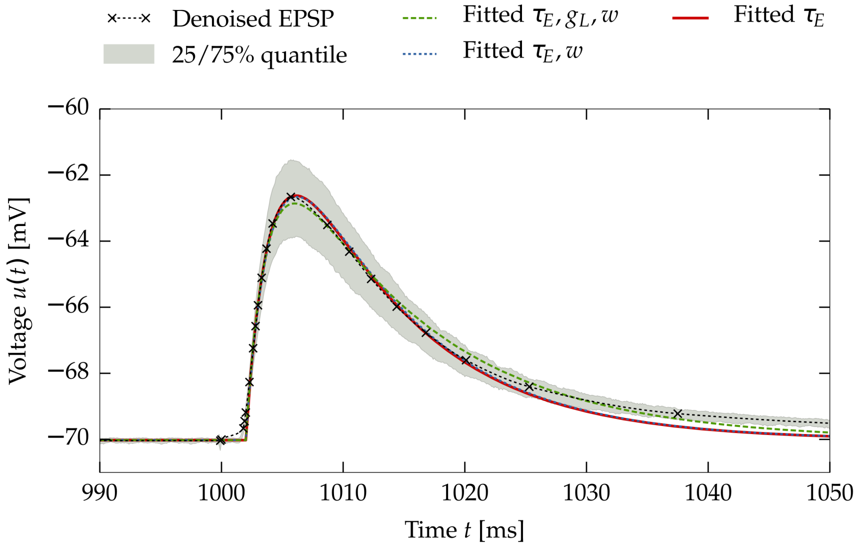

(b) LIF neuron simulation results with fitted parameters.

Figure S6: Analysis of 192 EPSP traces collected from all Spikey neurons for a single input spike at $t = 1000\,\mathrm{ms}$ and simulation results for a theoretical LIF neuron with fitted parameters. Figure (a) shows the 25/75% quantile, median, minimum/maximum and a denoised trace including its control points, (b) the simulation results for fitting LIF parameters to the denoised EPSP.
